# A Canonical Neural Map and Its Deviations Shape Parkinson’s Disease Phenotypes

**DOI:** 10.21203/rs.3.rs-8239383/v1

**Published:** 2026-01-12

**Authors:** Koorosh Mirpour, Amirreza Alijanpourotaghsara, Vibhash Sharma, Yvette Bordelon, Nader Pouratian

**Affiliations:** University of Texas Southwestern; UT Southwestern Medical Center; UT Southwestern Medical Center; UT Southwestern Medical Center; UT Southwestern Medical Center

**Keywords:** Parkinson’s disease, Deep Brain Stimulation, DBS, Beta Oscillation, Beta Burst, GPi, Motor Cortex, Cortical morphometry, cortical structure, electrophysiological biomarkers, neuroimaging, structural MRI, Gray matter thickness, Gray matter volume, Local field potential, LFP, ECoG

## Abstract

Parkinson’s disease (PD) presents a paradox: patients exhibit generally highly stereotyped network oscillations (excessive beta activity) yet patients manifest profound clinical heterogeneity and the literature is still sometimes inconsistent about the clinical significance of beta oscillations in PD. We resolve this discrepancy using multisite intracranial human recordings from the motor cortex and basal ganglia to define a robust, disease-specific “canonical spatio-spectral map” of network dysfunction. Crucially, we demonstrate that clinical variability is not random but is encoded by systematic, patient-specific deviations from this common template. The spatial regions exhibiting the highest inter-individual physiological variance specifically predict motor symptom severity, and data-driven clustering of these features reveals distinct clinical phenotypes. This framework establishes that individual deviations from a normative pathological map, often treated as noise, actually correlate with clinical symptoms. We provide a unified model of PD pathophysiology, linking the canonical beta signature to individual phenotypic diversity, and offer a quantitative blueprint for the development of personalized, circuit-specific therapeutic strategies.

## Introduction

One of the most consistently and well-documented electrophysiological findings in PD is the presence of excessive beta frequency oscillations (13–35 Hz) within and across cortical and subcortical basal nodes of the motor network (Brown et al., 2001; Malekmohammadi, Shahriari, et al., 2018). This manifests not only as elevated local power within the basal ganglia and cortical motor areas (Kuhn et al., 2008; Weinberger et al., 2006), but also as abnormally strong long-range coherence, between both the subthalamic nucleus (STN) and globus pallidus and the motor cortex (Malekmohammadi, AuYong, et al., 2018; Malekmohammadi, Shahriari, et al., 2018; Weinberger et al., 2006; Williams, 2002). These beta oscillations are strongly correlated with motor impairment, particularly bradykinesia and rigidity, and are attenuated by effective treatments such as levodopa administration and deep brain stimulation (DBS) of the STN or globus pallidus internus (GPi) (Kuhn et al., 2008; Malekmohammadi, Shahriari, et al., 2018; Ray et al., 2008). Despite the compelling evidence for pathological beta oscillations as a unifying hallmark of PD, the field is confronted by a fundamental paradox when viewed against the profound clinical variability of the disease.

PD is not a monolithic entity; it manifests with varying symptom profiles as well as heterogeneous responses to treatments (Mulroy et al., 2024; Von Coelln & Shulman, 2016; Wüllner et al., 2023). PD has long been parsed into clinical subtypes, such as tremor-dominant (TD), rigid-akinetic, and postural instability and gait difficulty (PIGD) phenotypes, which are associated with distinct patterns of neurodegeneration and clinical prognosis (Asch et al., 2020; Assogna et al., 2018; Jankovic et al., 1990). More recently, data-driven approaches have identified distinct “biotypes” based on brain atrophy, clinical profiles, progression rates, and other biology-driven multivariate datasets, formalizing the observation that PD follows multiple trajectories (Q. Chen et al., 2023; Deng et al., 2024; J. Wang et al., 2025; L. Wang et al., 2020; Zhang et al., 2019). This challenges the field to reconcile a highly stereotyped pathophysiological signature with the diverse clinical reality observed within and across individual patients.

In addition to clinical variability, while the association between beta activity and akinesia/rigidity is a central tenet of the field, even this relationship has been inconsistent across studies. Some investigations report robust positive correlations between beta power or coherence and motor impairment scores, such as the Unified Parkinson’s Disease Rating Scale Part III (UPDRS III) (Kato et al., 2015; Lofredi et al., 2023; Malekmohammadi, AuYong, et al., 2018; Van Wijk et al., 2016). However, other studies find only modest or non-significant associations (Litvak et al., 2011; Pardo-Valencia et al., 2024). Meta-analyses of non-invasive resting-state recordings have highlighted these mixed results, with some describing higher beta activity in PD patients, some lower, and some no difference compared to controls (Norouzi et al., 2025). This inconsistency may be partly attributable to methodological differences, medication status, or small sample sizes, but it also strongly suggests the presence of genuine and clinically meaningful biological variability. For instance, different motor symptoms appear to correlate with distinct oscillatory signatures; while akinesia and rigidity are linked to the beta band, resting tremor is more consistently associated with activity in the theta band (4–8 Hz), but not beta (Asch et al., 2020).

To reconcile these inconsistencies, we propose and provide evidence for a unifying framework that conceptualizes PD electrophysiology not just as a generalized state of “excessive beta,” but as a canonical spatio-spectral map. We hypothesize that this map represents a stable, common denominator of network dysfunction shared across the disease population—a pathological blueprint defining the core parkinsonian state. The consistent observation of this general pattern across numerous studies, methodologies, and patient cohorts supports the existence of such a stereotyped template of network dysfunction (Berendse & Stam, 2007; R. Chen et al., 2022; Gerster et al., 2025; Norouzi et al., 2025; Swann et al., 2015). We further posit that clinically meaningful variability is not random but is systematically encoded as quantifiable, patient-specific deviations from this canonical spatio-spectral map. While an individual’s neural patterns may closely resemble the population average, it is the specific magnitude and spatial location of their deviations from the norm that define each individual’s unique symptom profile and illness severity. The regions showing the greatest inter-individual variance in neural data often overlap with those most strongly correlated to individual symptom severity, suggesting they serve as clinically informative “hotspots.” Furthermore, data-driven clustering of these deviation features reveals subgroups with distinct clinical profiles, confirming that variability in biomarker expression encodes meaningful differences in motor symptomatology.

## Methods

### Study Participants

This study was approved by the Institutional Review Boards of both the University of California, Los Angeles (UCLA) and UT Southwestern Medical Center (UTSW). A primary cohort of patients diagnosed with idiopathic PD and a control cohort of patients diagnosed with ET were recruited across two sites (UCLA and UTSW). All participants provided written informed consent in accordance with the Declaration of Helsinki. DBS implantations and target selection were based solely on clinical grounds and indications, based on recommendations of an interdisciplinary clinical team. The PD cohort includes 75 individuals with PD (46 with GPi and 29 with STN DBS). For controls, we evaluated intracranial signals from 17 individuals with ET who underwent thalamic DBS implantation. This allows for the differentiation of neurophysiological patterns specific to PD pathophysiology from more general phenomena that could be associated more generally with movement disorders or the invasive intraoperative recording environment itself.

### Clinical and Demographic Assessment

Prior to surgery, diagnosis of isolated ET or PD was confirmed by a movement disorders neurologist (VS and YB). Motor symptom severity in those with PD was quantified using UPDRS III, in a practically-defined “off” medication state. Total UPDRS III score was used as a global index of motor severity to investigate broad relationships between biomarker characteristics and overall disease state. We also employed a more granular analysis using a vector of UPDRS III sub-scores, including measures for bradykinesia, rigidity, and tremor, further delineated by body side (e.g., ipsilateral Rigidity and contralateral Rigidity) to probe for associations between distinct neurophysiological subtypes and specific clinical phenotypes or symptom profiles. In addition to motor scores, key demographic data, including patient age and the number of years since diagnosis, were also considered. A summary of participant characteristics is provided in Table 1.

### Electrode Placement and Localization

**Figure 1:**
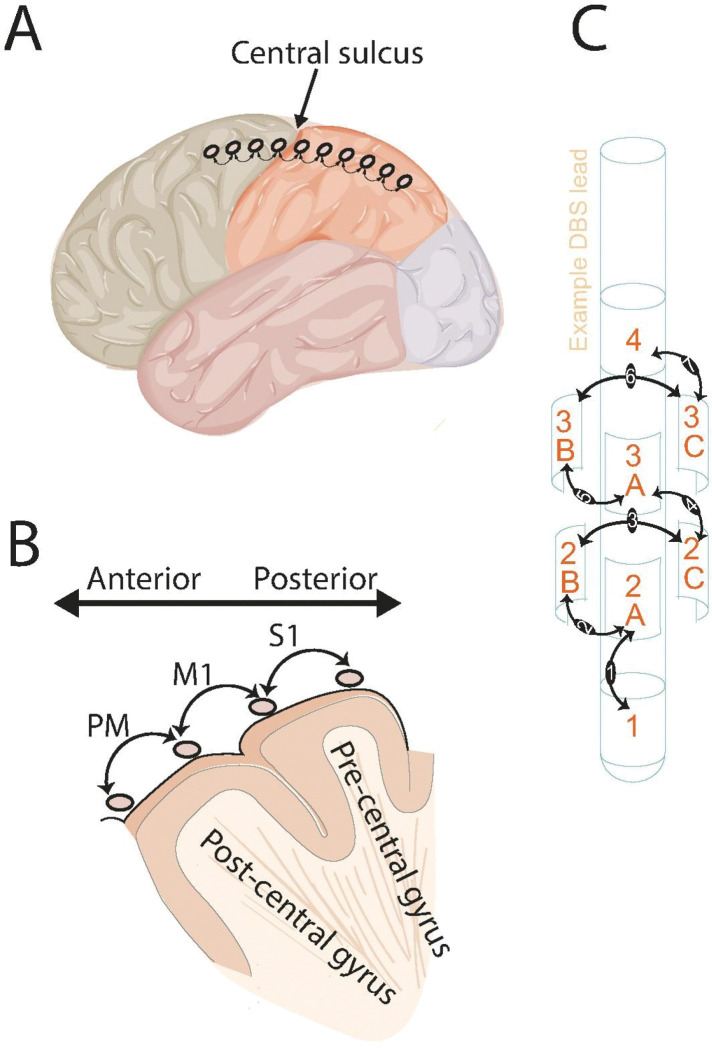
Intraoperative data acquisition setup and bipolar referencing scheme. Local field potentials were analyzed using a bipolar configuration, where the signal for each channel was calculated as the voltage difference between two adjacent contacts. **(A)** A subdural electrocorticography (ECoG) strip electrode array, containing eight contacts, is shown placed on the cortical surface. The array is positioned to span the central sulcus (CS), providing simultaneous coverage of the primary motor cortex (M1) and primary somatosensory cortex (S1). The curved arrows show the bipolar post recording re-referencing montage. **(B)** The schematic illustrates the terminology of bipolar re-referencing montage used for cortical signals. Three primary bipolar channels were defined based on their position relative to the central sulcus: PM (anterior to the CS), M1 (directly overlying the sulcus) , and S1 (posterior to the sulcus). **(C)** An example of a clinical deep-brain stimulation (DBS) lead used for subcortical recordings is depicted and bipolar analysis pairs.

All neurophysiological data were acquired intraoperatively during the stereotactic placement of permanent DBS leads. In each patient, a sterile, single-use subdural ECoG strip electrode array containing eight 4-mm platinum-iridium disc contacts spaced 1 cm apart (AdTech Medical) was temporarily placed on the surface of the sensorimotor cortex. The strip was positioned through the same burr hole created for DBS implantation, with a goal of spanning the central sulcus (CS), providing coverage of both pre-central (motor) and post-central (somatosensory) gyri (Fig. 1A). Commercially available clinical DBS leads, each featuring four or eight macroelectrode contacts arranged across four distinct depths were used for subcortical recordings, allowing for sampling of neural activity along the ventral-to-rostral axis of the target nucleus. Depending on the pre-determined surgical plan for each patient, the lead was implanted in either the GPi or the STN, based on intraoperative microelectrode recordings with kinesthetic testing and macrostimulation testing for both therapeutic benefits and adverse effects.

The three-dimensional coordinates of ECoG electrode contacts were determined using an established semi-automatic pipeline that integrates intraoperative imaging with the patient’s preoperative anatomical scans. This process is facilitated by a custom-built MATLAB interface (Randazzo et al., 2016). Each patient’s pre- and post-operative computed tomography (CT) scans was coregistered to their preoperative T1-weighted (T1w) anatomical magnetic resonance imaging (MRI) using the normalized mutual information method in Statistical Parametric Mapping (SPM12; Wellcome Centre for Human Neuroimaging, London, UK). The preoperative MRI was then processed through the recon-all pipeline in Freesurfer (version 7.3.2; http://surfer.nmr.mgh.harvard.edu/) to generate detailed 3D reconstructions of the pial surface and the corresponding cortical hull. Intraoperatively, two-dimensional (2D) lateral fluoroscopic images were acquired to visualize the ECoG electrodes in relation to the skull and brain. Within the localization interface, these 2D fluoroscopic images were then aligned with the 3D surface reconstructions. This crucial 2D-to-3D projection was guided by common anatomical and surgical landmarks visible in both imaging modalities, including the curvature of the calvaria, the trajectory of DBS leads, and the contact points of the stereotactic frame with the skull. This procedure allowed the precise location of each electrode contact to be mapped onto the individual’s cortical surface. The projected electrode positions were then manually verified and adjusted for accuracy using the visualization interface. The final three-dimensional coordinates for each contact were subsequently exported in the subject’s native anatomical space.

DBS electrode contacts were localized using the Lead-DBS toolbox (v3.0) (Neudorfer et al., 2023). Postoperative 1-mm axial CT scans were coregistered to preoperative MRI sequences, including T1w, T2-weighted (T2w), and Fast Gray Matter Acquisition T1 Inversion Recovery (FGATIR) images. The patient’s data was then normalized to Montreal Neurological Institute (MNI) standard space using the Advanced Normalization Tools (ANTs) library (Tustison et al., 2021), a process that inherently corrects for postsurgical brain shift. Following automated reconstruction, electrode trajectories were manually refined to ensure precise anatomical placement. The final coordinates for each contact were extracted in both the subject’s native space and MNI space. This methodology is consistent with that used in our previous studies (AuYong et al., 2018; Malekmohammadi, AuYong, et al., 2018; Malekmohammadi, Shahriari, et al., 2018).

### Signal Acquisition and Processing

LFPs were recorded from all cortical and subcortical electrode contacts. Data were acquired during a continuous 3-to-5-minute period of rest, during which patients were awake and alert but were explicitly instructed to remain as still as possible, to relax, and to refrain from making any voluntary movements or speaking. Neurophysiological recordings were conducted with a single scalp reference electrode at a sampling rate of 2,400 Hz at UCLA and 1,375 Hz at UTSW. At UCLA, recordings utilized the BCI2000 v3 system using g.Tec, g.USBamp 2.0 amplifier, and at UTSW, the NeuroOmega system was used.

The initial preprocessing pipeline included removal of 60 Hz line noise, lowpass and high pass filter at 2 and 120 Hz,and discarding segments with electrical or movement artifacts, following previously published procedures (AuYong et al., 2018). Artifact segments were identified based on criteria such as abnormally high power spectral values, excessive harmonics, and rapid voltage changes. Artifact removal was automated through full-wave rectification, calculation of the first derivative, and application of a five-point median filter. Segments where the first derivative exceeded five standard deviations (SDs) of the subject’s entire recording were considered artifacts and replaced by linear interpolation using data from 2 ms before and after the artifact.

To enhance spatial specificity and reject common-mode noise and volume-conducted signals from distant sources, the original monopolar recordings were converted into a bipolar montage. For the ECoG analysis, three bipolar channels were defined, including premotor cortex (PM,voltage difference between two anterior electrodes to the CS), primary motor cortex (M1, between the two contact across the CS), or primary somatosensory cortex (S1, posterior to the CS) (Fig. 1A–B). For the 8-contact DBS leads, this resulted in 7 bipolar channels, where the signal for each channel was the voltage difference between two adjacent contacts (e.g., channel 1 = contact 1 - contact 2, Fig. 1C). All subsequent spectral analyses were performed on these bipolar signals.

### Calculation of LFP Power and Spatio-spectral Power Maps

Power spectral density (PSD) was estimated using Thomson’s multitaper method (Thomson, 1982) with 1-second consecutive, non-overlapping time windows across a frequency range of 4 to 110 Hz. A frequency bandwidth of ± 2 Hz and three tapers were used. To control inter-individual variability in baseline power, each segmented spectrum was normalized to the total power of the signal for each condition, excluding line noise and its harmonics. This normalization resulted in values expressed in arbitrary units (a.u.).

To isolate periodic neural oscillations from the confounding aperiodic, 1/f-like component of the power spectrum, we parameterized and removed the aperiodic background for each calculated power spectral density (PSD) estimate.

The aperiodic component of each spectrum was modeled as a function of the form:

$$P(f)=b-log\left(k+f^{\chi }\right)$$

where P is power in logarithmic units, f is frequency, b is the broadband power offset, χ (chi) is the aperiodic exponent, and k is the knee parameter defining the bend in the aperiodic fit. This model was fit to each PSD in semi-log space (logarithmic power, linear frequency) across a specified frequency range of the analysis.

Following fitting of the aperiodic model, the resulting 1/f-like component was removed from the original power spectrum. All subsequent analyses of oscillatory power were performed on these aperiodic-adjusted spectra unless otherwise specified. This method allows for a more accurate quantification of true oscillatory activity, independent of broadband spectral shifts (Donoghue et al., 2020). Average power was calculated within specific frequency bands—alpha (8–11 Hz), low beta (12–20 Hz), and high beta (21–35 Hz) using the multitaper PSDs.

### Creating Spatio-spectral Coherence Maps

To quantify the degree of functional connectivity or phase consistency between cortical and subcortical regions, magnitude-squared coherence was calculated between pairs of ECoG (x) and DBS (y) signals. Given the large number of possible ECoG-DBS channel pairings (e.g., 7 ECoG channels × 7 DBS channels = 49 pairs), a targeted data reduction strategy was employed to focus the analysis on the most robustly connected pathways. This “maximum coherence pairing” approach was implemented as follows:

1. **For analyzing cortical coherence patterns**: For each of the 7 ECoG channels, coherence was calculated each of the 7 subcortical DBS channels. The single DBS channel that yielded the highest average coherence value within the beta band (12–35 Hz) was identified. The full coherence spectrum from this “maximal” pair was then selected to represent the cortico-subcortical connectivity for that specific cortical location.
2. **For analyzing subcortical coherence patterns**: The process was inverted. For each of the 7 subcortical DBS channels, coherence was calculated with all 7 ECoG channels. The single ECoG channel that produced the maximal beta-band coherence was identified, and the spectrum from this pair was used to represent the connectivity for that subcortical location.

This approach allowed for the creation of interpretable 2D spatio-spectral coherence maps, analogous to the power maps, where the y-axis represented the location of the primary channel (cortical or subcortical), the x-axis represented frequency, and the color intensity represented the coherence magnitude of the maximally connected pair.

Average power and coherence from the patient cohort were projected onto a 3D cortical surface model using a previously described approach (Kubanek & Schalk, 2015). Spatially smoothed activity maps for each electrode were generated by applying a Gaussian decay function over the cortical mesh, centered at each electrode location. Individual patient data were co-registered to a standardized cortical surface, enabling visualization and group-level representation of activation patterns. This technique provides anatomically grounded and spatially continuous representations of cortical activity, facilitating direct visual comparison across participants. All projections were computed using the parameters described in the original implementation unless otherwise specified.

#### Statistical Analysis

A comprehensive, multi-layered statistical framework was used to rigorously test the study’s hypotheses regarding the significance, consistency, specificity, and clinical relevance of the identified spatio-spectral patterns.

### Statistical Significance of Group-Average Patterns

To determine whether the group-average spatio-spectral patterns were statistically robust, a non-parametric permutation test was conducted. For each signal (e.g., cortical power, GPi-cortical coherence), the spatial labels (i.e., the electrode assignments) of each individual’s spatio-spectral map were randomly shuffled. This process was repeated 1,000 times $\left(N_{\mathrm{perm}}=1000\right)$ to generate an empirical null distribution of group-average maps that would be expected under the hypothesis of no true spatial organization. The true, unshuffled group-average map ($X_{\mathrm{true}}$) was then compared to this null distribution on a pixel-by-pixel basis. For each pixel, a Z-score was calculated by comparing the true value to the mean ($\mu_{\text{null}}$) and standard deviation ($\sigma_{\text{null}}$) of the 1,000 permuted values:

$$Z=\frac{X_{\mathrm{true}}\;-\;\mu_{\mathrm{null}}}{\sigma_{\mathrm{null}}}$$

Two-tailed p-values were derived as:

p=2[1-Φ(|Z|)]

where Φ(⋅) denotes the cumulative distribution function of the standard normal distribution.

To address the significant multiple comparisons problem inherent in analyzing these high-dimensional maps, a stringent two-stage correction procedure was applied. First, all p-values from the Z-tests were corrected using the Benjamini-Hochberg (BH) method to control the False Discovery Rate (FDR). The p-values were ranked in ascending order: p(1),p(2),…,p(m). The largest rank k was found such that its corresponding p-value, p(k), satisfied the following condition for an FDR level of α = 0.05:

$$p_{\left(k\right)}\leq \frac{k}{m}$$

Second, a cluster-based thresholding, referred to as “blob correction,” was implemented. This neurophysiologically-motivated approach posits that true effects should exhibit spatial and spectral contiguity. Therefore, a spatio-spectral pixel was only considered definitively significant if it was part of a contiguous cluster (or “blob”) of at least two adjacent electrodes × 3 Hz of consecutive bins, where every pixel within the blob had an FDR-corrected p-value less than 0.05.

### Consistency of Patterns Across Individual Subjects

A multi-pronged analysis was performed to quantify the consistency of the group-level patterns across individuals.

1. **Direct Population Comparison**: A one-sample signed-rank test was used at each spatio-spectral pixel to test if the values from individuals were significantly different from the population average. A low percentage of significant pixels across the map (e.g., < 14%) was interpreted as high consistency between individuals and the group average.
2. **Confidence Interval Analysis**: A 95% confidence interval (CI) was computed from the population data for each pixel of the spatio-spectral maps. For each individual, the percentage of their spatio-spectral data points that fell within this population-derived 95% CI was calculated. This percentage was significantly higher for the real data compared to spatially shuffled control data, as assessed by a paired t-test or signed-rank test, confirming that individual patterns were well-contained within the bounds of the population pattern.
3. **Correlation Analyses**: Two types of correlation were calculated to assess pattern similarity. First, the Pearson correlation coefficient (rho) was computed between each individual subject’s spatio-spectral map and the grand average map. Second, a cross-correlation matrix was generated by computing the Pearson correlation between the maps of all possible pairs of subjects. For all biomarkers, both sets of correlations were significantly skewed towards high positive values (mean rho > 0.5), indicating a substantial and statistically significant shared pattern across the cohort.

### Specificity of Cortical Power Patterns to Parkinson’s Disease

To establish the disease specificity of the observed cortical power map, a direct comparison was made between the PD and ET cohorts. A non-parametric Wilcoxon Rank Sum test was performed at each pixel of the spatio-spectral power map to identify regions with statistically significant differences in power between the two disease groups. The resulting map of p-values was then subjected to the same rigorous FDR and cluster-based “blob” correction procedure described previously to isolate significant clusters of activity on the cortical map that were unique to the PD cohort. To further confirm that the patterns of neural activity were significantly different between the two cohorts, we conducted a multivariate pattern analysis (MVPA). This approach treats the entire data matrix for each individual as a high-dimensional pattern, thus avoiding the statistical pitfalls of mass-univariate testing (Wilcoxon Rank Sum) and correction for multiple comparisons.

For each individual, the matrix of neural activity (channels X frequencies) was flattened into a single feature vector. A linear support vector machine (SVM) classifier was then trained to distinguish between subjects from the ET and PD cohorts based on these feature vectors. The classifier’s performance was evaluated using a 10-fold cross-validation procedure to ensure the model could generalize to unseen data and was not overfitted to the sample. This procedure yields a single mean classification accuracy, representing how well the distinct patterns of neural activity can differentiate the two groups.

To assess the significance of the classification accuracy, we performed a non-parametric permutation test. The group labels were randomly shuffled 1,000 times, and the entire 10-fold cross-validated classification was repeated for each shuffle. This process generated a null distribution of accuracies that would be expected by chance if there were no systematic difference in neural patterns between the groups. The p-value was calculated as the proportion of permutations that resulted in an accuracy greater than or equal to the accuracy obtained with the true labels. An alpha level of p < 0.05 was considered statistically significant.

### Analysis of Inter-Individual Variability and Correlation with Motor Impairment

This analysis aimed to test the hypothesis that the neurophysiological features that are most variable across the PD population are also the ones most relevant to clinical symptom severity. To this end, a two-step process was undertaken. First, to map inter-individual heterogeneity, the variance of the biomarker value across all subjects was calculated for each pixel in the spatio-spectral maps. A Z-test against variance maps from shuffled data was used to identify regions of significantly high variability. Second, to identify clinically relevant features, the biomarker value at each pixel was correlated (Pearson correlation) with each individual’s total UPDRS III score. The resulting maps of variance and clinical correlation were then qualitatively compared to identify areas of overlap.

### The relationship between electrophysiology Maps and Symptom Scores

To determine if the observed patterns of cortical activity were associated with clinical symptoms, a multiple regression analysis was conducted. This analysis used normalized spectral power as the dependent variable, with years since diagnosis, total UPDRS III score, and its sub-scores for ipsilateral and contralateral bradykinesia, rigidity, and tremor serving as the independent factors. To quantitatively model the multivariate relationship between the significant spatio-spectral electrophysiology features and the clinical measures, we employed Partial Least Squares (PLS) regression. PLS regression is designed to identify latent variables (LVs) that maximize the covariance between two sets of variables—in this case, electrophysiological data and clinical scores.

The electrophysiological input variables for the PLS model consisted of the top 10% of the spatio-spectral power and coherence maps that showed the highest variance among subjects. The clinical variables were the same as those used in the multiple regression analysis. To test whether the inter-individual electrophysiology variability correlates with clinical symptomology, we ran the PLS analysis for bottom 10% of the map with least variability among subjects and compared the results with top 10%.

To validate the model and prevent overfitting, a permutation test (10000 iterations) was performed by randomly shuffling the assignment of clinical scores to patients for each electrophysiological measure. An LV from the original analysis was considered significant if its correlation coefficient exceeded the 95th percentile of the null distribution generated from the shuffled data. Using the following formula:

$$p_{\mathrm{perm}\;}=\frac{\sum_{i=1}^{N}\left(\left|r_{\mathrm{perm}}^{\left(i\right)}\right|\geq \left|r_{\mathrm{obs}}\right|\right)}{N}$$

where

$p_{\mathrm{perm}}$ represents the p-value obtained from the permutation tests, i denotes the permutation iteration, $r_{\mathrm{perm}}^{\left(i\right)}$ is the correlation coefficient from the permuted data, and $r_{\mathrm{obs}}$ is the observed correlation coefficient from the original dataset.

To adjust for multiple comparisons, we employed the False Discovery Rate control using the Benjamini-Hochberg procedure using the method mentioned above. We rejected all null hypotheses for which the p-values were less than or equal to their corresponding threshold.

### Unsupervised Clustering of Spatio-spectral Biomarkers

To explore distinct neurophysiological subtypes within the PD population, an unsupervised clustering analysis was performed for each biomarker. First, a low-dimensional version of the spatio-spectral map was created using Principal Component Analysis (PCA). The optimal number of clusters (k) was then estimated using the Gap statistic method (Tibshirani et al., 2001) testing k from 1 to 10 from the first n principal components, which explained 80% of the data variation. The Gap statistic provides a quantitative criterion for selecting k by comparing the within-cluster dispersion of the data to that of a null reference distribution without inherent clusters. It calculates a single metric value by comparing a clustering solution to a simulated reference distribution that has the characteristics of the input points but lacks any clusters. Given a set of clustering solutions for the same data set, each with a different number of clusters k (from 1 to 10), the optimal solution has the highest gap value. Following this, patients were grouped into clusters using the k-means algorithm, based on their individual spatio-spectral patterns. The subsequent clinical analyses were performed using both the optimal k suggested by the Gap statistic and, for comparison, a fixed k = 2.

### Association of Neurophysiological Clusters with Clinical Symptom Profiles

To determine if the data-driven neurophysiological clusters corresponded to distinct clinical phenotypes, a sequential statistical approach was used.

1. **MANOVA**: A Multivariate Analysis of Variance (MANOVA) was performed first. This powerful test treated the cluster assignment as the independent variable and the entire vector of UPDRS III sub-scores as the multivariate dependent variable. A significant MANOVA result (p < 0.05) indicated that the overall *pattern* or *profile* of motor symptoms differed significantly between the neurophysiological clusters, even if no single symptom did.
2. **ANOVA**: Following a significant MANOVA, a series of post-hoc univariate Analyses of Variance (ANOVAs) were conducted. Each individual UPDRS III sub-score was tested separately to identify which specific symptoms were driving the significant multivariate effect observed in the MANOVA.

### Robustness of Cluster-Symptom Relationships

A major challenge in clustering analysis is the potential for results to be sensitive to the algorithm’s initial conditions. To rigorously assess the stability and robustness of the identified relationships between biomarker clusters and clinical profiles, a large-scale simulation was performed. The entire analytical pipeline—k-means clustering (for k = 1 to 10) followed by MANOVA and ANOVA—was repeated 1,000 times, each with a different random initialization. The primary outcome of this simulation was the percentage of the 1,000 iterations that yielded a statistically significant result (p < 0.05) for the MANOVA and for each individual ANOVA. A high percentage was interpreted as strong evidence for a robust, replicable relationship between a biomarker-defined subtype and a specific clinical symptom profile, independent of the stochasticity of the clustering algorithm.

## Results

### Spatio-spectral Patterns of Cortical Neural Oscillations in PD

**Figure 2:**
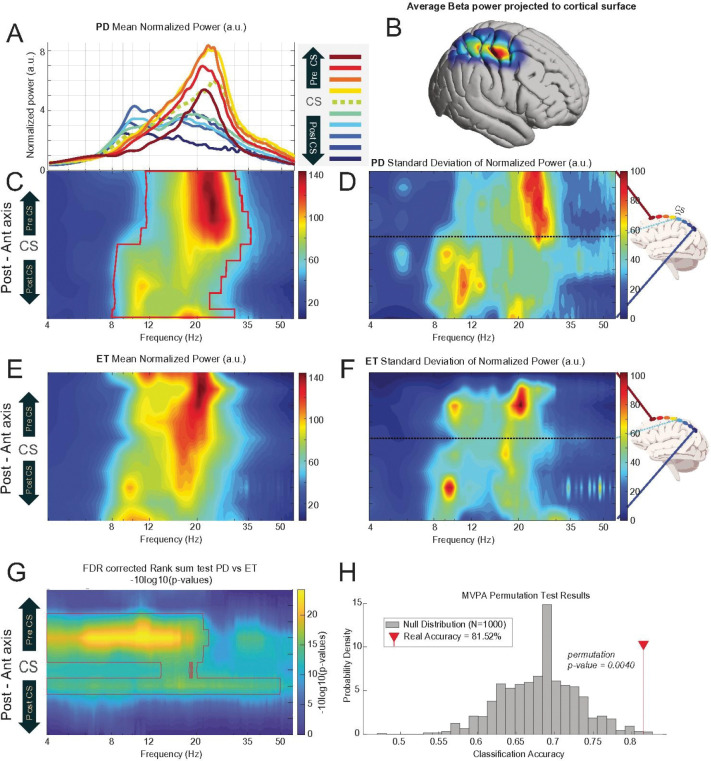
Canonical Spatio-spectral Pattern and Statistical Significance of Cortical LFP Power in PD and ET. **(A)** Mean normalized power spectra for PD patients, grouped by electrode location relative to the central sulcus (CS). **(B)** Spatial projection of mean beta power in PD. **(C, D)** Mean and standard deviation spatio-spectral power maps for the PD cohort. (E, F) Corresponding mean and standard deviation maps for the ET cohort. **(G)** Statistical map (FDR-corrected Rank Sum test) highlighting significant differences between the PD and ET power maps. **(H)**Multivariate pattern analysis (MVPA) classification results, showing significant accuracy (81.52%, p=0.004) in discriminating between the two cohorts based on their overall neural patterns.

Individuals with PD demonstrate specific spatio-spectral patterns of neural oscillations (Fig. 2A). Two primary peaks of power were consistently observed: a prominent peak within the beta frequency range, centered around 20–23 Hz, which was maximal anterior to the central sulcus (in motor cortices) and a secondary peak in the alpha frequency range, centered around 9–11 Hz, primarily posterior to the central sulcus. Furthermore, a smaller beta peak (~ 16–17 Hz), was identified in the most posterior ECoG contacts (Fig. 2A). The spatial distribution of the average beta cortical power, when projected onto a standard brain atlas, confirmed a concentration of activity predominantly over the motor cortex (Fig. 2B). To ensure the statistical robustness of the observed spatio-spectral patterns at the population level. For cortical power, the pattern was significantly different from a random distribution (permutation Z-test, BH corrected p < 0.05), particularly in anterior motor areas across a broad beta range, while in posterior sensory areas, significant deviations were primarily noted in the low beta and alpha band (Fig. 2B and C).

To investigate whether the observed cortical power maps were specific to PD. We employed a Wilcoxon Rank Sum test was used to compare the cortical power patterns between the PD (Fig. 2C–D) and ET (Fig. 2E–F) cohorts. This comparison revealed significant differences in key areas, highlighting distinct spots of high variance in PD patients (e.g., low beta power in motor and sensory regions, Fig. 2C–F). After applying False Discovery Rate (FDR) and blob correction, this analysis confirmed a significant group difference localized to pericentral electrodes (Fig. 2G).

While this mass-univariate approach successfully identified where the maps differed, it is not optimized to determine if the two patterns are significantly different when considered as a whole. To address this, we performed an MVPA. For this analysis, each subject’s data matrix was flattened into a single feature vector, and a linear support vector machine (SVM) classifier was trained to distinguish subjects from the PD and ET groups. The MVPA revealed a significant difference in the overall patterns of neural activity. The SVM classifier was able to distinguish between the groups with a mean cross-validated accuracy of 81.52%. This performance was significantly above the chance level established by a non-parametric permutation test (1,000 permutations, p value = 0.004, Fig. 2H).

### Subcortical LFP Power in Basal Ganglia

**Figure 3:**
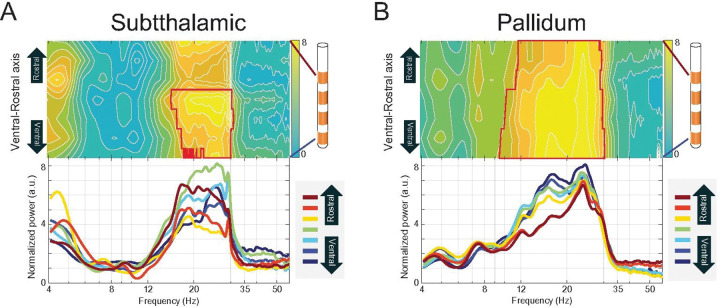
Canonical Spatio-spectral Patterns of Subcortical LFP Power in the Subthalamic Nucleus and Globus Pallidus Internus. Group-average spatio-spectral power maps and corresponding power spectra are shown for local field potentials (LFPs) recorded from Deep Brain Stimulation (DBS) leads. Power was calculated using Thomson’s multitaper method, normalized, and adjusted for the aperiodic 1/f-like component. The spatio-spectral map (top) and individual bipolar channel spectra (bottom) for **(A)** the **Subthalamic Nucleus (STN)**and **(B) Globus Pallidus internus (GPi)**, Power is plotted as a function of frequency (x-axis) and anatomical location along the ventral-to-rostral axis of the DBS lead (y-axis). In both panels, the red contours on the heatmaps delineate spatio-spectral regions where the observed power was significantly different from a null distribution generated by 1,000 spatial permutations (p<0.05, False Discovery Rate and cluster-corrected).

In the STN, consistent high beta band activity was evident across all contacts, extending from deep (ventral) to superficial (rostral) locations (Fig. 3A). Similarly, the GPi demonstrated consistent high beta activity across recordings sites. A notable distinction in the GPi was an increased peak in the low beta range observed specifically in the ventral leads, a feature not apparent in the more rostral GPi leads (Fig. 3B). STN power pattern showed significant deviation from random, with a patch of spatio-spectral significance observed in the middle to ventral STN contacts within the high beta frequency range (Fig. 3A, red outline). In the GPi, power patterns also significantly deviated from random; ventral GPi leads showed significance in both low and high beta frequencies, whereas the two most rostral GPi leads exhibited significant differences confined to the high beta band (Fig. 3B, red outline).

### Cortico-Subcortical Coherence

**Figure 4:**
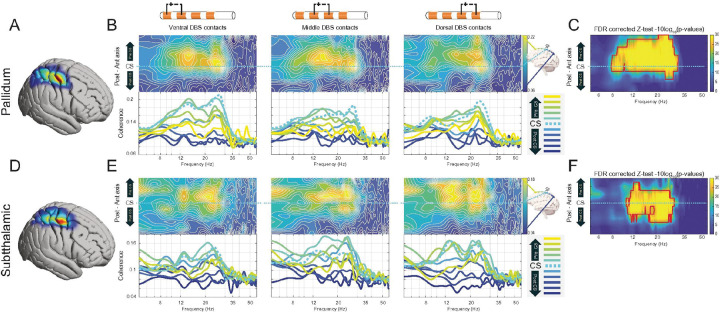
Spatio-spectral Characterization and Statistical Significance of Cortico-Subcortical Coherence. The figure details the group-average magnitude-squared coherence between cortical ECoG and subcortical DBS signals in the PD cohort. **(A & D)**Cortical surface projections illustrating the spatial distribution of average coherence within the 8–25 Hz frequency band for the **(A) Pallidum (GPi)**and **(D) Subthalamic Nucleus (STN)**. **(B & E)** Spatio-spectral coherence maps and corresponding spectra for **(B) GPi-cortical** and **(E) STN-cortical** pathways. **Heatmaps:** Coherence is plotted as a function of frequency (x-axis) and cortical ECoG channel location along the posterior-to-anterior axis (y-axis). Each map represents the coherence for a given cortical channel paired with the single subcortical channel depicted at the top of the column. The columns stratify the analysis by the location of the subcortical contacts on the DBS lead (Ventral, Middle, Dorsal). **Line Plots:** The plots below each heatmap show the coherence spectra for individual cortical channels, with colors corresponding to their anatomical position. **(C & F)**Statistical significance maps for the group-average coherence patterns shown for the **(C) GPi** and **(F) STN**. The color at each spatio-spectral pixel represents the p-value from a Z-test comparing the true group-average coherence to a null distribution generated from 1,000 spatial permutations. Values are plotted on a logarithmic scale (−10log10(p)). The analysis includes a two-stage correction for multiple comparisons, using the Benjamini-Hochberg method to control the False Discovery Rate (FDR) followed by a cluster-based correction.

The average cortico-subcortical coherence within the 8–25 Hz range, when mapped onto the cortical surface, showed peak activity immediately anterior to the central sulcus (i.e., motor cortex) for both GPi-cortical and STN-cortical coherence (Fig. 4A, D). Spectral analysis revealed that increased beta band coherence was a consistent feature of both STN-cortical and GPi-cortical interactions, characterized by a strong high beta peak (Fig. 4). A more pronounced low beta coherence peak was discernible in cortico-GPi coherence involving ventral and middle GPi bipolar contacts (Fig. 4B, left and middle column), and in cortico-STN coherence involving dorsal and middle STN bipolar contacts (Fig. 4E, middle and right column).

Regarding cortico-subcortical coherence, a significant patch of spatio-spectral frequency coherence between the cortex and GPi was observed in cortices anterior to the central sulcus (i.e., motor cortices) within the 8–35 Hz range (alpha and beta bands) using Z-tests on 1000-iterations shuffling, with p-values corrected for multiple comparisons through BH False Discovery Rate and blob correction (see methods), considering p < 0.05 (Fig. 4C). Similarly, motor cortices showed significant coherence with the STN in the 12–35 Hz (beta) range (Fig. 4F).

**Figure 5:**
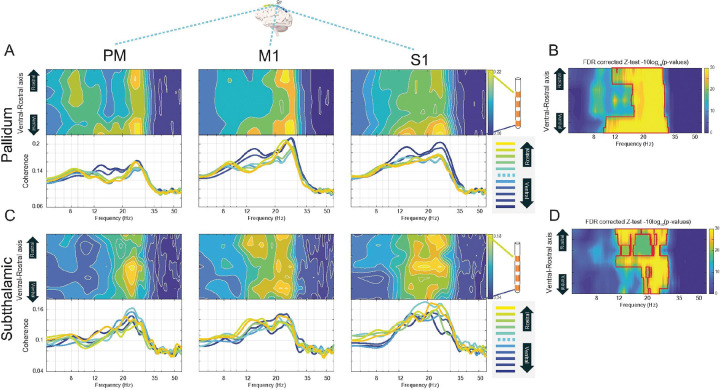
Spatio-spectral Characterization of Subcortical Coherence with the Cortex. This figure presents the group-average cortico-subcortical coherence from the perspective of the subcortical DBS leads. The data reduction strategy was inverted from that in Figure 4; for each subcortical bipolar channel, coherence was calculated against three central cortical channels, pre-CS, CS, post-CS. **A & C.** Spatio-spectral coherence maps and corresponding spectra for the **(A) Globus Pallidus internus (GPi)** and **(C) Subthalamic Nucleus (STN). Heatmaps:**Coherence is plotted as a function of frequency (x-axis) and subcortical channel location along the ventral-to-rostral axis (y-axis). The columns stratify the analysis by the location of the cortical ECoG contact (pre-central, central, and post-central sulcus). **Line Plots:** The plots below each map show the coherence spectra for individual subcortical channels, with colors corresponding to their anatomical position on the DBS lead. **B & D.**Statistical significance maps for the group-average coherence patterns shown for the **(B) GPi** and **(D) STN**. The color at each spatio-spectral pixel represents the p-value from a Z-test comparing the true group-average coherence to a null distribution generated from 1,000 spatial permutations. Values are plotted on a logarithmic scale (−10log10(p)). The analysis includes a two-stage correction for multiple comparisons, using the Benjamini-Hochberg method to control the False Discovery Rate (FDR) followed by a cluster-based correction.

When coherence was further analyzed stratified by cortical location (S1, M1 and PM, Fig. 5), high beta coherence with both STN and GPi was also evident across all these cortical regions, while low beta peaks emerged in some specific cortico-subcortical pairings (Fig. 5A, C). For GPi, significance was predominantly in the high beta range, with the exception of wider significance in two ventral and dorsal leads (Fig. 5B). STN coherence was significant in the beta range (Fig. 5D).

### Consistency of Spatio-spectral Patterns

To evaluate the consistency of population-level patterns across individuals, we used a one-sample Wilcoxon signed-rank test to compare the distribution of individual values against the population average at each point on the spatio-spectral maps. This analysis revealed no significant differences between individual data and the population average for any point on the maps of cortiucal power, GPi power, STN power, cortical-GPi coherence, or cortical-STN coherence (Benjamini-Hochberg corrected p < 0.05). We further calculated the percentage of subjects whose patterns were significantly different from the population average at any given spatio-spectral point. This percentage remained low across all biomarkers: <12% for cortical power, < 13% for GPi power, < 14% for STN power, and < 14% for both cortical-GPi and cortical-STN coherence. These low percentages are consistent with the expected rate of false positives (Type I errors), and this percentage approached zero when data were clustered into 2-contact by 3 Hz bins for blob correction (the percentage of subjects showing a 2-contact by 3 Hz significantly different from the population average).

**Figure 6:**
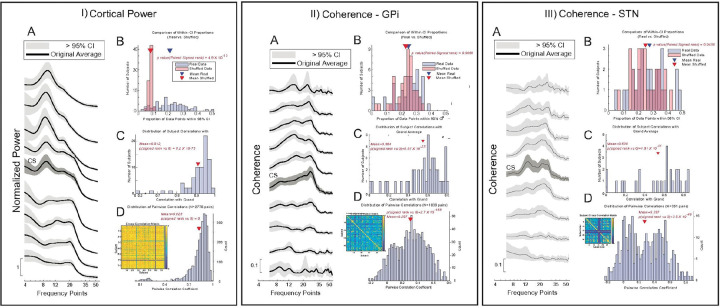
Consistency and Heterogeneity of the Canonical Cortical Power Pattern. The analysis is shown for **(I)**Cortical Power, **(II)** Cortex-GPi Coherence, and **(III)** Cortex-STN Coherence. **(A)** Grand average patterns (black line) with 95% confidence intervals (gray shade). **(B)** Histograms showing the proportion of data points falling within the 95% CI is significantly greater for real data (red) than for shuffled data (blue). **(C)** Distributions showing strong correlations between individual subject patterns and the grand average. **(D)**Pairwise correlation matrices (inset) and distributions showing weaker, more variable correlations between different subjects, highlighting individual heterogeneity.

Further analyses supported these findings. The average power and coherence for each individual was largely contained within the 95% confidence interval (CI) derived from the population (Fig. 6A). In all cases, there was a significantly higher percentage of observed data points falling within this CI compared to shuffled data (Wilcoxon signed-rank test, p = 4.9×10^−13^ for cortical power, p = 0.0056 for GPi coherence, and p = 0.0436 for STN coherence; Fig. 6, panels BB). Moreover, individual patterns were strong correlated with the grand average pattern (Pearson’s ρ > 0.5 for all comparisons; Fig. 6C, with mean correlations of 0.912 for cortical power, 0.554 for GPi coherence, and 0.538 for STN coherence).

In contrast to the high individual-to-average similarity, cross-correlations between the spatio-spectral patterns of different individuals showed greater variability (inset matrixes in Fig. 6D). Although the distribution of cross-subject correlation coefficients was significantly different from zero (all p < 0.001, Fig. 6D), the coefficients themselves were moderate for power (ρ ≈ 0.5 for cortex, STN, and GPi) and weak for coherence (ρ < 0.3) (Fig. 6D insets). These results suggest that while each individual’s neural activity aligns with a common population-level pattern, substantial heterogeneity exists between individuals, particularly in the coherence patterns.

### The relationship between electrophysiology Maps and Symptom Scores (UPDRS III)

**Figure 7:**
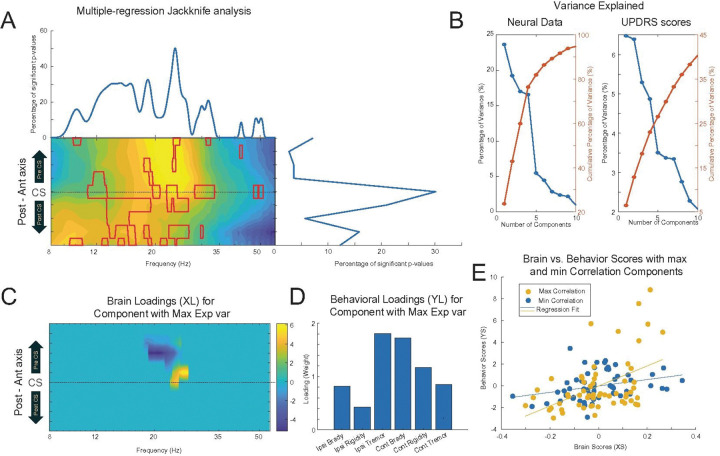
Relating Cortical Power Patterns to Clinical Symptom Severity using Multiple Regression and Partial Least Squares (PLS) Analysis. This figure illustrates the multi-stage analytical approach used to identify and model the relationship between spatio-spectral features of cortical power and motor symptom scores from the UPDRS Part III. **(A)** A preliminary **multiple regression analysis** identifying clinically relevant spatio-spectral regions. The heatmap shows the canonical spatio-spectral power map, while the line plots on the top and right show the percentage of significant correlations (p<0.05, corrected) between power at each pixel and the full vector of UPDRS III sub-scores. This analysis demonstrates that the strongest clinical associations are concentrated in the beta-band over sensorimotor areas. **Panels (B-E)**show an illustrative example of the **Partial Least Squares (PLS) regression**used to model the multivariate relationship between the top 10% of high-variance neural features and the clinical scores. **(B)** Scree plots showing the cumulative percentage of variance in the neural data (left) and the UPDRS scores (right) explained by the first 10 latent variables (LVs), or components, derived from the PLS model.

To determine if patterns of cortical activity in PD were associated with clinical symptoms, we conducted a multiple regression analysis using normalized spectral power as the dependent variable and clinical symptom scores as independent factors. The results revealed that the distribution of significant correlations coincided with the primary spectral and spatial features of the underlying cortical activity. Specifically, the percentage of significant p-values (p < 0.05, corrected for multiple comparisons) peaked within the low and high beta-frequency bands. Spatially, these correlations were concentrated around the electrodes overlying the sensorimotor cortex, directly corresponding to the frequency range and location of maximal power observed in the ECoG data (Fig. 7A). This trend was also apparent in subcortical power and coherence maps (data not shown) and motivated a more detailed subsequent analysis.

While the multiple regression analysis demonstrated a general association, it aggregated the effects of all clinical variables, thereby precluding the quantification of specific relationships between distinct spatio-spectral features and individual symptom sub-scores. To address this limitation, we employed Partial Least Squares (PLS) regression to quantitatively model the multivariate relationship between the electrophysiological features that showed high inter-individual variability and the clinical measures. An illustrative example of a single PLS iteration on cortical power and UPDRS III scores is presented in Fig. 7B–E. The analysis decomposes the relationship into a set of latent variables (LVs), showing the variance explained by the first 10 LVs (Fig. 7B), the weights of the LVs for power and UPDRS scores (Fig. 7C–D), and the correlations between the resulting power and clinical LV pairs (Fig. 7E).

To validate the model, we performed a permutation test (10,000 iterations). An LV from the original analysis was considered significant if its correlation coefficient exceeded the 95th percentile of the null distribution generated from the shuffled data. To test the hypothesis that cortical regions with high inter-individual electrophysiological variance are most representative of clinical status, we ran this validated PLS analysis on two distinct datasets: spatio-spectral features from regions with significant inter-individual variance and those from regions without.

The results consistently supported our hypothesis. LVs derived from high-variance regions of the cortical map explained a significantly higher percentage of variance in both electrophysiological measures (power and coherence) and UPDRS III sub-scores compared to LVs from low-variance regions (Table 2). A notable exception was the subthalamic nucleus (STN), for which we found no significant PLS correlations. Collectively, these findings demonstrate that clinical symptom scores are most strongly encoded within the peak spatio-spectral features of cortical activity, which are the loci of maximal electrophysiological power and inter-individual variability.

### Subcluster Analysis and Clinical Symptom Profiles

#### Optimal Number of Physiological Clusters

Although population-level biomarker patterns were consistent across subjects, we investigated the existence of data-driven subgroups within our cohort. The Gap statistic method was employed to determine the optimal number of clusters (k, ranging from 1 to 10) for each biomarker, based on their distinct spatio-spectral patterns. Power-based biomarkers, including cortical power, GPi power, and STN power, suggested optimal cluster numbers ranging from approximately 7 to 9 (cortical average k: 8.49; GPi: 8.81; STN: 7.11). In contrast, coherence-based biomarkers consistently indicated an optimal cluster number of 1.

#### Clinical Significance of Biomarker-Derived Clusters (MANOVA)

To determine if the biomarker-derived clusters were associated with distinct clinical phenotypes, a Multivariate Analysis of Variance (MANOVA) was performed. Using the UPDRS III sub-scores as response variables and cluster assignment as the independent factor, this analysis assessed whether the overall vector of motor scores differed across groups. Significant differences were found for clusters based on cortical power (BH-corrected p = 0.0001), GPi power (BH-corrected p = 0.0496), and STN power (p < 0.00001) (Table 3), indicating that biomarker-driven clusters can potentially explain the differences in clinical symptoms.

#### Differences in Individual UPDRS Sub-scores Across Clusters (ANOVA)

MANOVA captures the overall vector pattern of UPDRS sub-scores across data-driven groups. Post-hoc Analyses of Variance (ANOVAs) were used to test for differences in individual UPDRS sub-scores across the biomarker-derived clusters. Few individual sub-scores showed significant differences at an uncorrected threshold (p < 0.05) when using either the optimal number of clusters or a simplified binary split (k = 2) (Table 4). Critically, none of these individual findings survived FDR correction.

#### Robustness of Symptomology Patterns in Biomarker-Driven Clustering

The results of MANOVA and ANOVA analyses are highly influenced by the clustering method used and the initial clustering points. To evaluate the robustness of the relationship between clusters and clinical symptoms against the variability of the clustering algorithm, 1,000 iterations of clustering (for k = 2 − 10) were performed, each followed by MANOVA and ANOVA. The percentage of iterations yielding a significant MANOVA result (FDR corrected p < 0.05) was high for most biomarkers (62%–90%), indicating that biomarker-driven subgroups are robustly associated with distinct overall symptom patterns (Table 5). In contrast, the percentage of iterations yielding significant ANOVA results for individual sub-scores, total score, age, or years since diagnosis was generally lower. However, the UPDRS Total Score, when clustered by STN power, yielded significance in 72% of iterations. This suggests that while overall symptom patterns are robustly differentiated, the link to single UPDRS sub-scores is less consistent, with the relationship between STN power and total motor score being a notable exception. These results suggest that while the overall clinical profile differs across biomarker-defined groups, these differences are not consistently driven by any single motor symptom.

## Discussion

The pathophysiology of PD is represented by a highly consistent and statistically robust spatio-spectral signature of cortico-basal ganglia activity. This reproducible, disease-specific canonical pattern is characterized by elevated beta-band power in the pre-central motor cortex and strong beta-band coherence with the STN and GPi. Importantly, while individual patterns closely match the population average, individual deviations from this canonical map, particularly within the beta range, are not random but clinically relevant and correlate with motor symptom severity. Data-driven clustering of these spatio-spectral features reveals subgroups with distinct overall clinical profiles, indicating that variability in biomarker expression encodes meaningful differences in motor symptomatology.

### A Canonical and Specific Spatio-Spectral Map of Parkinson’s Disease

Our first hypothesis posited the existence of a statistically robust, canonical map of neural activity defining PD. Indeed, we confrimed a highly structured and significant group-average pattern characterized by prominent motor cortex beta power (~ 20–23 Hz) and strong beta-band coherence between the motor cortex and both the STN and GPi. Within the basal ganglia, both nuclei exhibited strong beta power, with the GPi showing a distinct low-beta peak in its ventral aspect not seen in the STN.

These findings align with and substantially extend previous work identifying elevated beta oscillations as a hallmark of PD and PD-related symptoms. By confirming both local beta power increases and long-range cortico-subcortical beta coherence, we reinforce the consensus that these features are central electrophysiological markers of the disease (Brown et al., 2001; Kuhn et al., 2008; Malekmohammadi, AuYong, et al., 2018). However, while prior studies most often focus on oscillatory behavior in one or two nodes in the motor network, we move beyond a general description of “excessive beta” to define a detailed, multi-dimensional atlas of parkinsonian network dysfunction. The statistical significance of these group-level patterns, established through permutation testing, confirms they are not artifacts of averaging but represent a true underlying spatial and spectral organization (Figs. 2–5). The consistency of this pattern across individuals (Fig. 6) solidifies its status as a canonical signature. This high degree of conformity to a central template suggests a shared, fundamental mechanism of network pathology common to most patients. The significant elevation of motor cortex beta power in the PD cohort compared to ET patients (Fig. 2) confirms that this spatio-spectral pattern is not a generic feature of movement disorders or the recording environment (intraoperative during DBS surgery) but is a specific neurophysiological signature of PD. Our disease-specific mapping addresses the ongoing debate over the specificity of beta biomarkers by demonstrating significant differences from ET, in agreement with reports distinguishing PD from other movement disorders (Gerster et al., 2025; Norouzi et al., 2025).

### Clinically Meaningful Heterogeneity: Reconciling Variability and Symptom Severity

While the motor network physiology across patients adheres to the same general pathological canonical map, each individual expresses a unique variation of it with substantial inter-individual variation (Fig. 6D, insets). This variation is not random. Rather, inter-individual variability is clinically meaningful. Deviations from the canonical pattern within circumscribed spatio-spectral “hotspots” is systematically associated with degree and pattern of clinical motor impairment. The proposed “canonical map + deviation” framework is further supported by identifying the strongest symptom correlations occurring in regions of maximal beta power.

This model reconciles the apparent paradox between stereotyped group-level pathophysiology and marked clinical heterogeneity in PD. For instance, beta power in motor cortical locations is both highly variable across patients and strongly associated with motor symptom severity. This directly supports our hypothesis that the most informative biomarkers for predicting an individual’s clinical state are found in the very spatio-spectral locations that differ most across the patient population. This provides a powerful explanation for the inconsistent correlations between beta activity and motor scores reported in the literature (Litvak et al., 2011; Pardo-Valencia et al., 2024; Norouzi et al., 2025). Studies may not have been able to identify strong associations because they averaged across entire frequency bands or anatomical regions, diluting the contribution of these specific, highly variable, and clinically relevant “hotspots.”

Further supporting the critical role of this canonical spatio-spectral map in driving clinical symptomatology, data-driven neurophysiological clusters across this map are associated with distinct multivariate clinical profiles, as demonstrated by the highly significant and robust MANOVA results (Table 5). However, these clusters did not neatly map onto individual UPDRS sub-scores (e.g., rigidity or bradykinesia) after correction for multiple comparisons. This suggests that the names of the classic clinical subtypes like “tremor-dominant” or “PIGD” may be imperfect reflections of the underlying neurophysiology. Instead, an individual’s place in the neurophysiological landscape appears to determine a unique *constellation* of motor deficits rather than the severity of a single symptom. The brain’s pathological state is high-dimensional, and its clinical expression is likewise a complex multivariate profile, not a simple scalar value. The robust link between STN power clusters and the total UPDRS score, however, highlights the central role of this nucleus in determining overall motor disability. Of note, clusters were defined by power-based biomarkers (cortical, GPi, and STN power), whereas coherence-based biomarkers did not. This suggests that the magnitude of local and distributed oscillatory power may be a more sensitive substrate for subtyping than the synchrony between regions, which appears as one single cluster of varying spectra.

### Implications, Limitations, and Future Directions

The canonical map framework offers a powerful new lens through which to view PD pathophysiology and can have significant implications for clinical practice. The canonical map serves as a normative atlas of the diseased state, providing a stable reference against which individuals can be compared. The patient-specific deviation map, in turn, acts as a personalized neurophysiological “fingerprint,” pinpointing the specific loci of greatest dysfunction for that individual. This approach provides the blueprint for personalized care. For example, in the case of DBS, rather than applying uniform stimulation parameters, future closed-loop DBS systems could selectively consider and target the spatio-spectral hotspots identified on a individual’s deviation map, potentially increasing efficacy while minimizing side effects.

This study has several limitations. The data were acquired intraoperatively during a state of rest, and it remains to be seen how these spatio-spectral maps evolve during active movement or in response to medication. Furthermore, our clinical correlations relied on the UPDRS III scale; future work should incorporate objective kinematic measures for a more fine-grained analysis. We also predict that inclusion of morphometric and structural connectivity maps derived from diffusion-weighted imaging can potentially enrich this framework, by providing additional metrics of individual anatomic variability. Finally, our analysis is correlational and cannot establish causality. Future studies should leverage this framework to track disease progression longitudinally, to investigate the neural correlates of non-motor symptoms, and, most importantly, to guide the development of next-generation, patient-specific closed-loop therapies.

This study resolves the apparent paradox between the consistent neurophysiological hallmarks of PD, variability of these biomarkers across individuals and across studies, and the heterogeneous clinical presentation of PD. We demonstrate that clinically-relevant information is encoded in patient-specific deviations from a canonical spatio-spectral maps, where the most variable neural features are the most predictive of symptom severity. The canonical map framework provides a unified model of PD pathophysiology and lays the quantitative foundation for a new era of personalized diagnostics and neuromodulation.

## Supplementary Material

Supplementary Files

This is a list of supplementary files associated with this preprint. Click to download.

• tablesmerge.pdf

• fig8.jpg

Tables

Tables 1 to 5 are available in the Supplementary Files section.

## Data Availability

The datasets analyzed during the current study are available in the Brain Integrated Resource for Human Anatomy and Intracranial Neurophysiology(B(RAIN2) repository, https://doi.org/10.18120/zmah-2816.

## Code availability

The underlying code for this study is not publicly available but may be made available to qualified researchers on reasonable request from the corresponding author.
